# BINge: Multispecies Ortholog Clustering for Differential Gene Expression Analyses

**DOI:** 10.1111/1755-0998.70137

**Published:** 2026-04-27

**Authors:** Zachary Stewart, Dimitri Perrin, Alexie Papanicolaou, Peter Prentis

**Affiliations:** ^1^ School of Biology and Environmental Sciences Queensland University of Technology Brisbane Queensland Australia; ^2^ Centre for Agriculture and Bioeconomy Queensland University of Technology Brisbane Queensland Australia; ^3^ School of Computer Science Queensland University of Technology Brisbane Queensland Australia; ^4^ Centre for Data Science Queensland University of Technology Brisbane Queensland Australia; ^5^ Hawkesbury Institute for the Environment Western Sydney University Sydney New South Wales Australia

**Keywords:** cross‐species, DGE, genomics, sequence clustering, transcriptomics

## Abstract

Differential gene expression (DGE) analysis enables researchers to investigate the link between gene expression and the phenotypic responses observed in organisms across time, experimental, or field conditions. Accurate quantification of gene expression is essential when performing DGE experiments, with a range of methods having been developed to enable the study of gene expression within a species. Quantifying differences in expression not just within but across multiple species can also be used to reveal the genetic mechanisms underlying phenotypic differences observed between species. Accurate quantification of gene expression across multiple species requires a suitable reference; it should include each species' own expressed transcripts to mitigate reference bias, with the orthology relationships of transcripts being used to facilitate comparison of expression at the gene level. Production of such a reference remains a challenge, despite its necessity for minimising bias during multispecies DGE analysis. Our software BINge specifically aims to address this need through use of a novel approach to modelling orthology which results in multispecies transcript clusters that accurately reflect their locus orthology. Evaluation experiments demonstrate the effectiveness of this approach over existing clustering methods which have not been designed for producing a reference suitable for multispecies DGE analysis. Source code and documentation for BINge are available from the GitHub repository at https://github.com/zkstewart/BINge.

## 1 Introduction

Differential gene expression (DGE) studies typically compare RNA samples from a single species across time or treatment conditions relative to controls. Sequencing reads are aligned to a reference genome or transcriptome to facilitate comparison of expression levels for a common set of genes (or in some cases, isoforms). Under such an experimental design, it is clear that a genome or transcriptome sourced from the species being studied should be used as a reference. Assuming that the genome or transcriptome assembly is of high quality, this will ensure that virtually all transcripts expressed by the studied organism are represented in the reference.

However, the choice of reference is less clear for a study comparing expression patterns across species. These studies often seek to discover whether differences in gene expression contribute to phenotypic differences between species. An appropriate reference should avoid reference bias by including the full diversity of transcripts across all species under study. To facilitate comparison of a common set of genes, the orthology relationships between these transcripts must be established (Julca et al. 2023). This idealized reference would thus enable unbiased read mapping to species‐specific transcripts within the reference, with subsequent aggregation of read counts based on orthology to yield gene‐level expression estimates for multispecies DGE comparisons.

A key challenge in creating this idealised reference is to perform a clustering process whereby each resulting cluster represents a single locus whose members consist solely of orthologous species‐specific transcripts. One approach to produce such clusters is to perform pairwise sequence alignments to measure their sequence similarity; bioinformatic tools which perform this include CD‐HIT (Li and Godzik 2006) and MMseqs2 (Steinegger and Söding 2017). If the similarity between two sequences exceeds a chosen threshold, they are deemed to be ‘related’ in some capacity and, as a graph, this can be represented through an edge connecting the sequence nodes. A problem with this approach is the difficulty or impossibility of identifying a similarity threshold value that will optimally produce the desired outcome for all datasets.

Other approaches attempt to explicitly model orthology. These include OrthoFinder (Emms and Kelly 2019) through its utilisation of phylogenetics, and SonicParanoid2 (Cosentino et al. 2024) with its assessment of protein domain architecture. These programs seek to identify ‘orthogroups’ consisting of genes inferred to have descended from a single ancestral gene in the last common ancestor of all species being analysed. This definition is broader than what is required for our idealised reference wherein each cluster should correspond to a single locus alone rather than a group of loci.

We have produced BINge (bin genes for expression analyses) to facilitate the creation of our idealised reference where each cluster aims to represent a single locus; we refer to this as being a ‘locus cluster’. BINge allows for a combination of reference gene models and/or *de novo* assembled transcriptomes from one or more species to be used as input. Orthology relationships between sequences are derived through a biologically inspired approach to clustering that makes use of one or more reference genome assemblies. BINge performs this clustering and produces aggregated read counts for each locus cluster that are amenable to downstream DGE analysis in R.

In this manuscript, we describe four experiments to validate the accuracy of BINge when producing locus clusters from a variety of real datasets. Our results indicate that BINge produces highly accurate locus clusters, unlike most existing clustering algorithms, which are not designed to achieve locus‐level clustering. Hence, BINge is uniquely suited for performing the clustering task necessary for a subsequent multispecies DGE analysis to take place.

### 1.1 Implementation Details

BINge is written in Python 3 and pipelines a process involving flexible input of data through to the output of locus clusters and, optionally, read counts associated with each locus cluster. Gene model coding DNA sequences and/or transcriptome sequences to be clustered can be provided from any number of species; these input sequences are henceforth referred to as transcripts. A key novelty of BINge is its reference‐based clustering, whereby input transcripts are clustered based on shared overlap of alignment to any number of reference genomes. This provides information regarding the locus—the physical location within a genome—where each transcript is likely to have been transcribed from. Ideally, each species represented in the input transcripts will have its associated reference genome provided; however, as we demonstrate later, the genome of one or more closely related species can be a suitable substitute.

Input transcripts are aligned against the reference genome(s) using GMAP (Wu and Watanabe 2005) to obtain their likely intron‐exon structures. The location where an exon alignment occurs is termed a ‘bin’; as a data structure, each bin is defined as a genomic region bounded by a start and stop coordinate which collects the identifiers of any transcripts which have an alignment within that genomic region. Through interpretation of the GMAP alignments, bins are ‘seeded’ along a genome to maintain a record of any transcripts whose exons originate from those locations in the genome. The seeding of bins can be performed *de novo* based on information obtained from GMAP alignment, or alternatively a process of ‘bin pre‐seeding’ can be performed. Pre‐seeding involves an initial assessment of a reference genome's gene model annotations to define the start and stop coordinates of each bin based on reference exon annotations. This allows for subsequent interpretation of GMAP alignment whilst ensuring that bins are positioned based on the reference annotations instead of the GMAP alignments which may not exactly recapitulate the annotated exon positions. In our later evaluations we compare the performance of *de novo* bin seeding and pre‐seeding to provide objective insight into its impact on clustering outcomes. The information recorded by these bins is used to cluster transcripts using a process referred to as ‘clustering by co‐occurrence’.

### 1.2 Clustering by Co‐Occurrence

**FIGURE 1:**
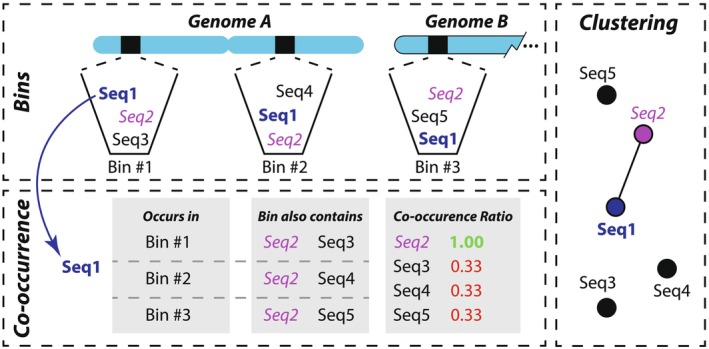
Diagram depiction of the clustering by co‐occurrence process. After bins have been seeded throughout all genomes, BINge iteratively loops through each sequence identifier and locates the bins it occurs within. In the figure, sequence 1 (Seq1) is selected and the bins it occurs within (#1, #2, and #3) are located. The co‐occurrence ratio for each other sequence that occurs in the same bins as Seq1 is calculated by tallying the number of times the other sequence is found divided by the total number of bins that the current sequence (Seq1) occurs within. In the example figure, Seq2 occurs within all three of the same bins as Seq1 and hence has a co‐occurrence ratio of 1. As a result, Seq1 and Seq2 will have an edge connecting them in the graph data structure used for clustering.

The algorithm for transcript clustering has been termed ‘clustering by co‐occurrence’. In this approach, each transcript is represented as a node, with edges being formed between transcripts which should be clustered together; the graph data structures used are implemented in Python's NetworkX library (Hagberg et al. 2008). The clustering process iterates over each transcript (the ‘current transcript’) and identifies any other transcripts which occur in a shared bin. For each ‘other transcript’, the co‐occurrence of the current transcript with the other transcript is measured as the number of times the two transcripts occur in a shared bin divided by the total number of bins that the current transcript occurs within (see Figure 1). This gives the proportion of times the current transcript occurs in a bin alongside the other transcript. If the co‐occurrence proportion is greater than or equal to a user‐specifiable threshold, an edge is formed between the transcript nodes. Note that while this ‘co‐occurrence proportion threshold’ is user‐configurable, we later describe our process for objectively deriving a default value which produces optimal results in almost all cases. At this stage, the components of the graph are filtered to remove transcripts from consideration which would otherwise induce erroneous clustering behaviour. These include fragments and chimeras, with their handling detailed in subsections below.

### 1.3 Fragment Removal

Fragmented transcripts, which often occur as single exons and hence are found in only a single bin, induce erroneous behaviour as they will always co‐occur with any other transcript that occurs in the same bin. To identify these fragments, we check for bidirectional co‐occurrence that is, we also calculate the co‐occurrence proportion of the ‘other transcript’ with the fragmented ‘current transcript’. We expect to observe a one‐sided co‐occurrence relationship wherein fragments will co‐occur with longer transcripts, but the longer transcripts will not co‐occur with the fragment. Transcripts which form a one‐sided co‐occurrence relationship in ≥ 50% of their total relationships can be removed as probable fragments.

### 1.4 Chimera Removal

Chimeras are identified by assessing each component within the larger cluster graph. Specifically, we expect that chimeras will occur as articulation points within a component whereby their removal would leave no edges connecting two or more subgraphs (see Figure S1). We note that the detection of chimeric transcripts using this method requires that a chimeric transcript must occur as a singleton in the dataset, as two or more identical or near‐identical chimeric transcripts would not provide the opportunity to locate the chimera as an articulation point. Nonetheless, when identified, these transcript nodes are removed.

### 1.5 Clustering Output

After fragment and chimera removal, components are output as ‘binned’ locus clusters. At this point, any transcripts from within the original inputs which failed to align against a reference genome will not have been included in the reference‐based clustering process and hence remain as ‘unbinned’ transcripts; these may represent real genes which are not present in any reference genomes or may instead be contaminants or erroneous transcript assemblies. Unbinned transcripts are clustered by BINge using either CD‐HIT or MMseqs2 with user‐specifiable parameters. Note that we provide default recommended parameters for this process which were objectively derived from analyses detailed later in this report.

### 1.6 Cluster Representatives and DGE Interrogation of Results

BINge streamlines the use of MMseqs2 to query clustered transcripts against a database of proteins such as the UniRef database (Suzek et al. 2015). It additionally pipelines the alignment of RNA‐seq data to transcripts using salmon (Patro et al. 2017) to quantify gene expression. Alignment and expression data for clustered transcripts can be used to select cluster representatives. And, when using the UniRef database, representative transcripts can be automatically functionally annotated by BINge with their gene names and Gene Ontologies (Ashburner et al. 2000). A tabular report can be generated to facilitate investigation of these results. Importantly, after salmon quantification has been performed, BINge can generate a pre‐configured R script to easily load raw transcript counts and tally these counts to the locus level using the tximport R package (Soneson et al. 2016).

## 2 Materials and Methods

We developed a custom evaluation process which compares clustering results against an original reference genome annotation (as a GFF3 file); the script for doing this (*evaluate_clustering.py*) is provided in BINge's code repository. Each protein‐coding locus from the GFF3 annotation, inclusive of its alternative splice isoforms, is treated as a true cluster group and is compared to a clustering result using several metrics including Rand Index (RI), Adjusted Rand Index (ARI), Normalised Mutual Information (NMI), and Adjusted Mutual Information (AMI). These metrics range from a value of 0 to 1, with increasing values indicating greater agreement between test (clustering output) and true (reference annotation) clusters. The ARI and AMI metrics are modified versions of the RI and NMI metrics, respectively, and are adjusted to account for chance. In practice, this means that random or simple clustering outcomes are penalised for example, where most clusters contain exactly one transcript.

This method of evaluation tests the ability of a clustering tool to take a deconstructed reference annotation (i.e., the gene model transcripts) and reconstruct a clustering outcome where the transcripts are grouped into their original loci. It is expected that splice isoforms such as those involving exon skipping as well as gene families arising from recent duplication events would pose difficulties for most clustering algorithms. These challenges may result in multiple loci being grouped into a single locus cluster, or alternative splice isoforms occurring as separate clusters. The chosen evaluation approach penalises these occurrences as they would introduce bias or inaccuracies that negatively affect a DGE analysis. Additionally, we scored the completeness of the cluster representative transcripts using compleasm (Huang and Li 2023). We expect that effective locus clustering should minimally reduce completeness scores and result in the number of output clusters being comparable to the number of loci found in the original reference annotation. Using these metrics, we sought to identify which clustering solution was best suited to produce locus clusters.

### 2.1 Evaluation of Single Species Clustering

The performance of software when clustering a single species was assessed to provide a baseline measurement of how effectively a clustering approach could take a deconstructed reference annotation and reconstruct the original gene annotations as clusters. Later assessments would include multiple species to see how clustering performance compares between these scenarios. Software used in this single species clustering evaluation included BINge (using GMAP version 2024‐11‐20) alongside pairwise sequence alignment programs including CD‐HIT (v4.8.1) and MMseqs2's cascaded clustering algorithm (commit fcf5260). We also tested corset (v1.09; Davidson and Oshlack 2014), which opts to use shared RNA‐seq read alignments to cluster transcripts. Inclusion of corset allowed for comparison of BINge to another software that also seeks to identify locus clusters.

#### 2.1.1 Data Selection

A range of species were selected to provide coverage of microbial taxa (bacteria, archaea, and fungi) as well as plants and animals. We specifically selected only intensively studied model organisms whose ‘gold standard’ annotations would provide the greatest opportunity to accurately test the performance of each clustering approach; note that the relatively understudied nature of archaea makes identification of an organism from this Kingdom which fits this criterion difficult. The selected species consisted of: microorganisms = *Candida albicans* (GCF_000182965.3; Muzzey et al. 2013), *Saccharomyces cerevisiae* (GCF_000146045.2; Engel et al. 2022), *Escherichia coli* (GCF_000008865.2; Hayashi et al. 2001), and *Haloferax lucentense* (GCF_029225785.1); plants = *Brassica rapa* (GCF_000309985.2; Zhang et al. 2018) and *Arabidopsis thaliana* (TAIR10; Lamesch et al. 2012); animals = *Danio rerio* (GCF_000002035.6; Howe et al. 2013), *Homo sapiens* (GCF_000001405.40; Schneider et al. 2017), and *Xenopus tropicalis* (GCF_000004195.4; Mitros et al. 2019).

As corset relies upon RNA‐seq alignment, we downloaded publicly available datasets for each species from the NCBI SRA (Bosseboeuf et al. 2023; Furman et al. 2020; Gao et al. 2024; Hurtig and van Hoof 2023; Wang et al. 2023) with no explicit approach taken other than to obtain recent paired end sequencing from control conditions and with high sequencing depth. Datasets selected are listed in Table S1. We chose not to evaluate corset on *E. coli* or *H. lucentense* as their lack of exons and isoforms makes them a poor fit for the clustering approach of corset.

#### 2.1.2 Parameterisation of Clustering Tools

To our knowledge, there has been no systematic investigation into how to best parameterise CD‐HIT or MMseqs2 to produce results akin to locus clusters. Hence, to fairly compare BINge against these programs, we first sought to find a combination of parameters which would most accurately result in their prediction of locus clusters. For CD‐HIT, we tested 160 combinations of parameters including sequence identity (‐c), short sequence coverage (‐aS), long sequence coverage (‐aL), and global (‐G 1) or local alignment (‐G 0). For MMseqs2 cascaded clustering, we tested 500 combinations of parameters including sequence identity (‐‐min‐seq‐id), coverage ratio (‐c), *E*‐value (‐e), and sensitivity (‐s); we used connected‐components clustering and did not test different modes as we encountered errors when performing set‐cover clustering. The optimal parameters for these tools were determined to be those which, on average, maximised the clustering metrics obtained when reconstructing the original gene annotations of the reference genomes.

We similarly wanted to identify what parameters provided BINge with optimal results. Specifically, we tested several ‘co‐occurrence proportion threshold’ values to ascertain what impact this had on clustering. We also ran BINge with and without bin pre‐seeding to see how this affected its results.

For corset, we used default parameters except where we turned off filtering of transcripts with low read counts (‐m 0). Corset only outputs transcripts which have at least one read alignment, which we chose not to penalise by omitting any absent genes from the true cluster groups during its evaluation.

#### 2.1.3 Ranking of Clustering Tools

Each clustering tool was ranked according to its maximisation of the clustering metrics. Only the results obtained after parameter optimisation (where relevant) were compared herein. We also measured the time taken for each tool to complete after a single run. Where clustering tools required time‐consuming setup that is, GMAP or salmon alignment, times are provided with and without those setup operations. The time taken to perform reference genome indexing with GMAP or salmon were not included. All tools were executed with eight threads available for any multithreaded operations on a high‐performance computing cluster.

### 2.2 Evaluation of Multispecies Clustering

The performance of software when clustering gene transcripts obtained from multiple species was assessed. This evaluation sought to validate the suitability of BINge for producing multispecies locus clusters and to compare the accuracy of its results against existing approaches. In addition to BINge and MMseqs2 cascaded clustering, this multispecies clustering evaluation included OrthoFinder and SonicParanoid2. We did not include corset as its approach is not suited to a multispecies clustering task. We also opted not to include CD‐HIT in any further comparisons after observing near‐identical performance to MMseqs2 in the single species clustering evaluation.

#### 2.2.1 Parameterisation of Clustering Tools

Our previous parameter optimisation of MMseqs2 was specifically intended for use during single species locus clustering. We did not assume these same parameters would be optimal for multispecies clustering. Hence, we ran 43 different combinations of sequence identity (‐‐min‐seq‐id) and coverage ratio (‐c) parameters. Results were reported only for the parameter combination that maximised the clustering metrics. For BINge, we made use of the optimised ‘co‐occurrence proportion threshold’ and omitted pre‐seeding from this test and all downstream tests based on results from the individual species clustering evaluation.

#### 2.2.2 Data Selection and Ranking of Clustering Tools

The reference genome and annotation of four citrus species were obtained from the Citrus Genome Database (www.citrusgenomedb.org). These included *Citrus reticulata* (MSYJ v1.0; Wang et al. 2018), *C. sinensis* (DHSO v3.0; Wang et al. 2021), *C. maxima* (Cupi Majiayou v1.0; Lu et al. 2022), and *C. hindsii* (S3y‐45 v2.0; Wang et al. 2022).

To calculate the clustering metrics for each species, we filtered clustering results to produce a subset where only a single species' transcripts were represented. The filtered clustering result could then be compared to the species' own reference genome annotation. Aside from not knowing what the true multispecies locus clustering result should be, this method allowed us to assess whether each individual species' loci were remaining accurately clustered among the larger set of multiple species' transcripts. We additionally obtained the longest transcript from each cluster as a representative and computed the completeness score of these representative transcripts using compleasm with reference to the BUSCO eudicots database (odb10; Simão et al. 2015).

### 2.3 Evaluation of Reference Genome Choice

As BINge is unique in its use of reference genomes to guide the clustering process, we sought to measure the impact that reference genome choice had on its clustering outcome. We wanted to observe any changes in performance that might occur when clustering a single species' gene transcripts with reference to (1) its own genome or (2) a related species' genome or (3) a combination of several related species' genomes. By making use of the same citrus genomes and annotations as in the multispecies genome evaluation, we had already obtained results for a fourth scenario wherein a species' own genome is used in combination with several other related species' genomes. Obtaining these results would provide a gradient in reference genome choice to reveal trends in how this choice affects clustering outcomes.

### 2.4 Evaluation of Evolutionarily Divergent Clustering

High‐quality reference genomes spanning a continuum of evolutionary distances were clustered to test the evolutionary limit of BINge's ability to accurately perform multispecies locus clustering. Genomes and annotations were sourced from NCBI RefSeq (O'Leary et al. 2016) for *H. sapiens* (GRCh38.p14; Schneider et al. 2017), *Mus musculus* (GRCm39; Chinwalla et al. 2002), *Rattus norvegicus* (mRatBN7.2; de Jong et al. 2022), and *D. rerio* (GRCz11; Howe et al. 2013). NCBI's RefSeq orthologous gene pairs as found in the ‘gene_orthologs.gz’ file was downloaded from the NCBI FTP site on January 17th, 2025. This file describes one‐to‐one pairs of orthologous genes for each species we surveyed in this evaluation. The file was parsed as a graph whose connected components were considered to represent true cluster groups for comparison with the clustering tool outputs. In addition to evaluating the clustering outcome for the entire set of gene transcripts, we also tested each pairwise combination of species to assess the performance of ortholog clustering across a variety of phylogenetic distances. Comparison of each individual species' results was performed against its own reference genome to facilitate a full pairwise heatmap display and provide visualisation of how single‐species clustering compared to pairwise species clustering performance. Completeness scoring with compleasm was performed with reference to the BUSCO vertebrata database (odb10). To assess how BINge handled this challenge relative to another clustering tool, we again ran MMseqs2 with 43 different combinations of identity and coverage ratio and reported only the optimal parameter combination's result.

## 3 Results

### 3.1 Optimisation of CD‐HIT and MMseqs2 Clustering

In general, optimal CD‐HIT clustering of animal and plant genomes was obtained when using a high identity cut‐off (‐c 0.95 or 0.98), local alignment (‐G 0), and when specifying the short and long sequence coverage ratios (‐aS and ‐aL) as 0.4. Clustering of microbial genomes was optimised by using an identity cut‐off of 0.98, global alignment (‐G 1), and the long sequence coverage set to ≥ 0.8 with the short sequence coverage being inconsequential. For MMseqs2, the assessed *E*‐value and sensitivity parameters were inconsequential for all species. An identity cut‐off of 0.98 provided optimal performance for all species. Animal and plant genomes clustered best with a coverage ratio of 0.4. Microbial genomes clustered best when the coverage ratio was set to ≥ 0.6 ( *H. lucentense* ) or 0.95 (*S. cerevisiae, C. albicans*, and *E. coli* ).

Overall, results suggest that locus clustering of animal and plant genomes should be performed using an identity score of 98%, coverage ratio of 40% for both the short and long sequence, and local alignment where applicable. Microbial genomes should use 98% identity and 80% coverage ratio for short and long sequences, with global alignment being used where applicable. These optimised parameters were used in the rest of this evaluation, with the full set of these results available in File S1.

### 3.2 Optimisation of BINge Clustering

Different values for the ‘co‐occurrence proportion threshold’ parameter resulted in only minor changes to clustering outcomes. When comparing a lower value of 0.33 to a higher value of 0.66, the largest difference in the number of output clusters was observed for *H. sapiens* . Specifically, a parameter value of 0.33 resulted in the prediction of 19,411 locus clusters and a parameter value of 0.66 instead resulted in 20,828 locus clusters; this difference of approximately 6.80% was the largest observed, with most species having a difference of 1.55% or less. The difference in cluster metrics was also small, although a ‘co‐occurrence proportion threshold’ value of 0.5 or 0.66 provided marginally better metrics for most species. We set a threshold value of 0.66 to be the default for BINge and used it in all subsequent tests. The full set of results for this evaluation are available in File S2.

### 3.3 Performance of Single Species Clustering

**FIGURE 2:**
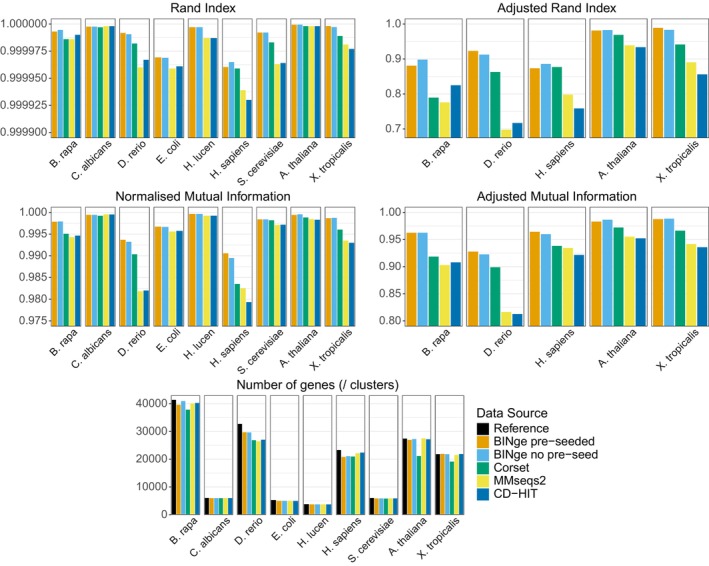
Cluster statistics obtained in the first test which evaluated BINge, corset, MMseqs2, and CD‐HIT when clustering gene sequences from four microbial and five plant or animal genomes. Adjusted Rand Index and Adjusted Mutual Information values are not shown for the four microbial taxa as values approximately equal to zero were obtained owing to these metrics' penalisation of simple or random clustering. Values closer to 1 indicate better performance in all cluster metrics. For the number of clusters, a value similar to the number of reference genes is ideal. *B. rapa* = *Brassica rapa* ; *C. albicans* = *Candida albicans* ; *D. rerio* = *Danio rerio* ; *E. coli* = *Escherichia coli* ; *H. lucen* = *Haloferax lucentense* ; *H. sapiens* = *Homo sapiens* ; *S. cerevisiae* = *Saccharomyces cerevisiae* ; *A. thaliana* = *Arabidopsis thaliana* ; *X. tropicalis* = *Xenopus tropicalis* .

Results for the single species clustering evaluation (Figure 2) indicate that BINge produces locus clusters with a high degree of accuracy, outperforming other tools for all species except *C. albicans* where MMseqs2 displays marginally better performance based on the NMI score (0.999541 versus 0.999433). In general, clustering tools perform comparably for the microbial genomes, likely owing to their simple nature. Plant and animal genomes provide a greater challenge, which results in larger differences in clustering metrics among the clustering tools. For example, the best performance for *H. sapiens* is seen in BINge (AMI = 0.964288), with the next best result seen for Corset (AMI = 0.938277). Overall, a trend is seen for animal and plant genomes whereby BINge performs best, followed by corset, then by either CD‐HIT or MMseqs2. Results also indicate that BINge performs comparably with or without bin pre‐seeding. Raw results for this evaluation are available in File S3.

**FIGURE 3:**
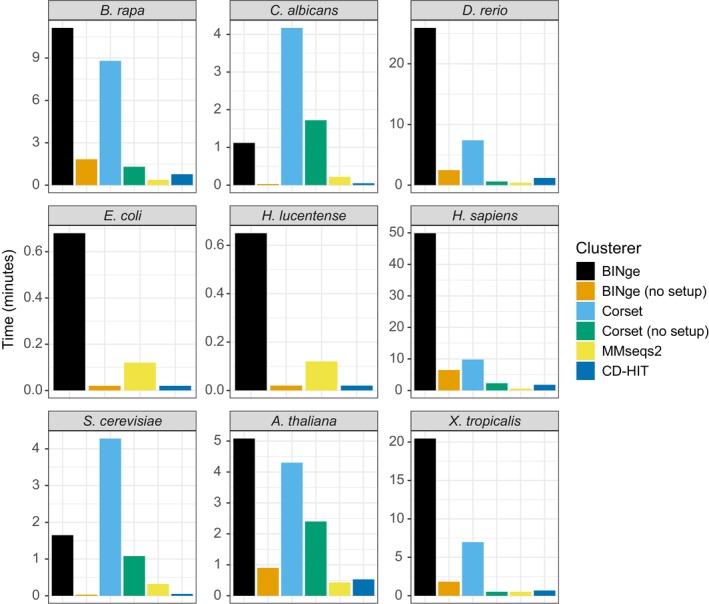
Time taken for each clustering program to complete on each of nine reference genomes. For BINge, the time taken is indicated for the whole pipeline inclusive of sequence extraction and GMAP alignment as well for when setup is not required that is, when only the clustering step is run. For Corset, the time is provided for the whole pipeline or when setup is not required that is, when salmon mapping has already been performed. Corset was omitted from analysis in *E. coli* and *H. lucentense* . *B. rapa* = *Brassica rapa* ; *C. albicans* = *Candida albicans* ; *D. rerio* = *Danio rerio* ; *E. coli* = *Escherichia coli* ; *H. lucen* = *Haloferax lucentense* ; *H. sapiens* = *Homo sapiens* ; *S. cerevisiae* = *Saccharomyces cerevisiae* ; *A. thaliana* = *Arabidopsis thaliana* ; *X. tropicalis* = *Xenopus tropicalis* .

With respect to program execution time (Figure 3), BINge is the slowest clustering tool in most organisms and takes up to approximately 50 min to cluster the gene transcripts of *H. sapiens* . Corset was the slowest tool when clustering *C. albicans* and *S. cerevisiae* due to relatively long salmon alignment times for their RNA‐seq data. MMseqs2 and CD‐HIT are the fastest clustering tools, with no organism taking MMseqs2 longer than 1 min to cluster. When excluding setup operations including GMAP and salmon alignment, we find that all tools complete clustering in relatively short time spans.

### 3.4 Performance of Multispecies Clustering

**FIGURE 4:**
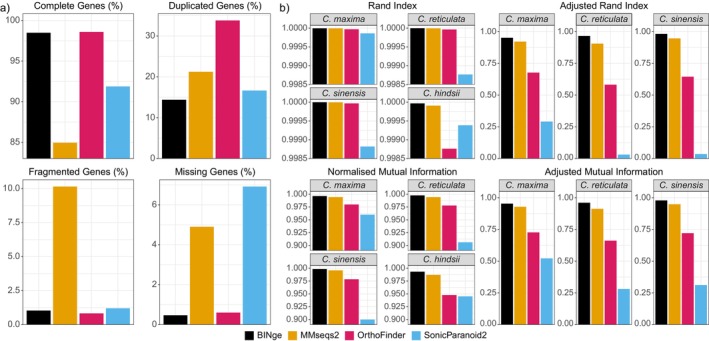
Cluster evaluation statistics obtained when using different methods to cluster the gene transcripts of *Citrus maxima* , *Citrus reticulata* , *Citrus sinensis* , and *Citrus hindsii*. (a) Completeness scores are shown for eudicotyledon genes expected to occur as single‐copy genes in almost all members of this phylogenetic clade. This indicates the percentage of genes which are full‐length (complete), as well as genes which are duplicated, fragmented, or missing for the set of cluster representative transcripts. (b) Cluster metrics are shown for each species by comparing the species' reference gene annotation against a subset of the clustering results where only that species' transcripts were represented. Results for *C. hindsii* gene transcript clustering are omitted from Adjusted Rand Index and Adjusted Mutual Information results as these values approached zero and were not informative for comparison.

The completeness scores obtained after clustering the four citrus species' gene model transcripts are depicted in Figure 4a. The highest completeness was found for OrthoFinder (98.58%) followed closely by BINge (98.49%). Although BINge shows marginally lower completeness than OrthoFinder, it has a greatly reduced duplication percentage despite having a higher number of predicted locus clusters (OrthoFinder = 31,667; BINge = 49,917); this might suggest that unique loci are being represented by BINge which are not factored into the completeness scoring. SonicParanoid2 produces results with a low duplication percentage and a small number of clusters (20,951), but this appears to come at the cost of having a high percentage of missing genes. MMseqs2 was parameterised with a sequence identity cut‐off of 0.95 and a coverage ratio of 0.4. Results obtained with these optimised parameters demonstrate the lowest completeness percentage despite having the highest number of clusters (56,843). Its elevated fragmented gene percentage also suggests that many fragmented genes are not clustering with their full‐length counterparts.

Clustering metrics for the multispecies evaluation are shown as Figure 4b. BINge outperforms all other tools for these metrics, with MMseqs2 displaying a lower but still comparable performance. Following this are OrthoFinder with a further decline in clustering metrics seen for SonicParanoid2. Note that ARI and AMI results for *C. hindsii* are all approximately equal to zero, making the metrics uninformative and hence these results are not shown. See File S4 for raw results of the multispecies clustering evaluation.

### 3.5 Performance Variation With Reference Genome Choice

**FIGURE 5:**
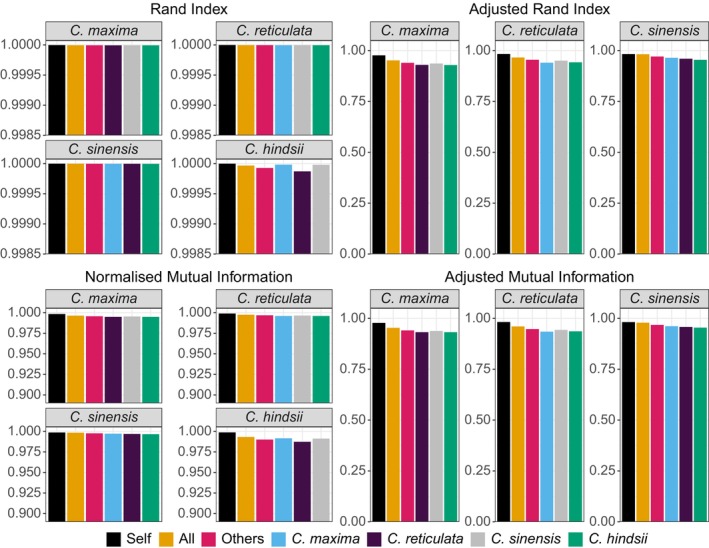
Cluster evaluation statistics obtained when using BINge to cluster the gene transcripts of *Citrus maxima* , *Citrus reticulata* , *Citrus sinensis* , and *Citrus hindsii*. Clustering was performed using different reference genome selections and their results are displayed with differently coloured bars. The label ‘Self’ refers to the evaluation statistics obtained when clustering a species (indicated in subplot headers with grey outline) using its own reference genome. ‘All’ refers to the outcome of clustering a species' gene transcripts using all four citrus reference genomes. ‘Others’ refers to the outcome of clustering when omitting a species' own genome and using only the other three species' reference genomes. The remaining bars show the result obtained when using the indicated species' genome as the only reference. Results for *C. hindsii* gene transcript clustering are omitted from Adjusted Rand Index and Adjusted Mutual Information results as these values approached zero and were not informative for comparison.

Clustering metrics obtained when clustering the four citrus species' gene model transcripts with different reference genome(s) are depicted in Figure 5. Clustering metrics are maximised when clustering a species using only its own reference genome. The second‐best result is obtained when clustering a species using all four citrus genomes as a reference. Clustering metrics continue to marginally decline when clustering with three related genomes as a reference, with the lowest metric values seen when clustering using only one related genome. As before, ARI and AMI results for *C. hindsii* are all approximately equal to zero and are hence not shown. See File S4 for raw results of this evaluation.

### 3.6 Performance for Evolutionarily Divergent Clustering

BINge clustering for the entire set of vertebrate gene transcripts resulted in a total of 69,597 clusters with ortholog clustering metrics of ARI = 0.858 and AMI = 0.912. MMseqs2, when optimally parameterised with sequence identity of 0.92 and coverage ratio of 0.6, produced 86,229 clusters with ARI = 0.696 and AMI = 0.841. Completeness scoring shows that BINge and MMseqs obtain near‐100% completeness with a very high duplication percentage (i.e., > 94%).

**FIGURE 6:**
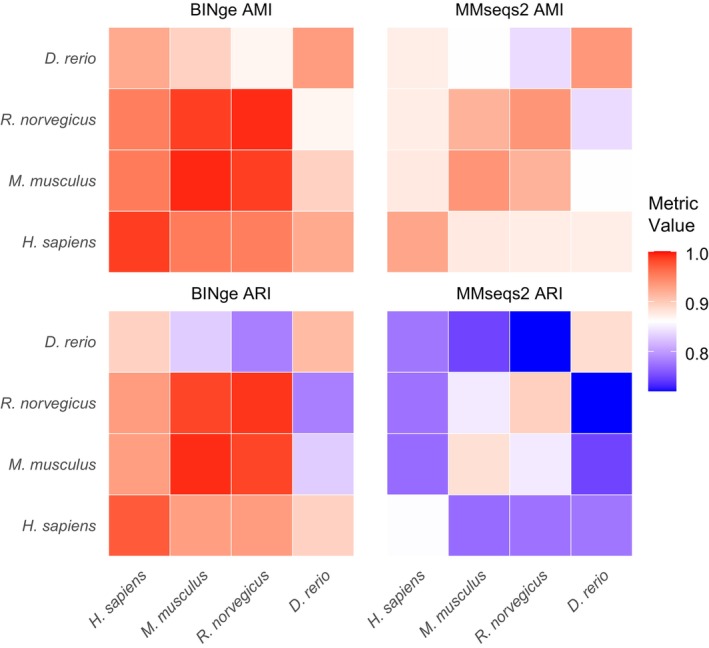
Adjusted Mutual Information (AMI) and Adjusted Rand Index (ARI) cluster evaluation statistics obtained when using BINge and MMseqs2 to cluster the gene transcripts of *Danio rerio* (zebrafish), *Rattus norvegicus* (brown rat), *Mus musculus* (house mouse), and *Homo sapiens* (human). The accuracy of clustering is shown for each pairwise comparison of species with respect to their one‐to‐one gene ortholog pairs. Self‐comparison indicates the accuracy of clustering when compared to the species' own reference genome.

The accuracy of pairwise species clustering outcomes is indicated as a heatmap in Figure 6. Orthologous loci in pairwise comparisons of *H. sapiens* , *M. musculus* , and *R. norvegicus* are discovered well by BINge, with the minimum observed value for ARI being 0.930 and for AMI the minimum value being 0.951. Clustering of *D. rerio* 's gene transcripts appears to be challenging, with self‐comparison and its pairwise comparisons with other species obtaining the lowest values. The same overall trends are observed for MMseqs2 clustering, albeit with lower clustering metric values when compared to what BINge obtains. Full clustering metrics and completeness scores for this evaluation are provided in File S5.

## 4 Discussion

Our evaluations sought to test each clustering tool's ability to produce ‘locus clusters’, which we consider to be achieved when gene transcripts are grouped according to their shared physical (orthologous) locus in a genome. Most clustering tools assessed herein do not specifically seek to produce locus‐level clusters for example, OrthoFinder which aims to identify ‘orthogroups’ or MMseqs2 which does not aim to produce any particular type of clustering outcome. BINge was specifically designed to accomplish the task that our evaluations tested for, and it subsequently excels in the designed evaluations. The inclusion of corset in our single species clustering evaluation was to provide a fair point of comparison since it also aims to perform locus‐level clustering. The congruity between corset's goals and the design of our evaluation also resulted in corset obtaining high values for the clustering metrics, which were comparable to (although generally slightly lower) than what BINge obtained. With corset as a point of reference, we believe that we can fairly conclude that BINge produces locus clusters with a high degree of accuracy. Given their similarity in performance, a researcher intending to perform single species locus‐level clustering may elect to choose one software or the other depending on data availability; corset results are contingent on high‐quality RNA‐seq data availability, whereas BINge instead relies upon high‐quality and phylogenetically close reference genome availability. However, we note that only BINge can handle multiple species.

The multispecies evaluation demonstrates how the intent underlying BINge's implementation results in superior multispecies clustering outcomes when compared to tools which are not designed for this use case. BINge is the only tool which maintains high gene completeness, low gene duplication, and a close relationship between transcript clusters and their true loci according to clustering metrics. This type of clustering is essential for multispecies DGE analysis to take place without introducing reference bias associated with the use of only one species' reference genome or transcriptome; instead, sequencing reads can map to the constituent transcripts of a BINge cluster and be tallied at the locus level. BINge facilitates this process, streamlining the introduction of raw cluster read counts into an R environment for subsequent DESeq2 normalisation and analysis (Love et al. 2014).

BINge also appears to be relatively insensitive to reference genome choice when the selected genome(s) come from the same genus as the species whose transcripts are being clustered. Cluster metrics are optimised when using only a species' own genome, which is expected as this most closely represents a scenario where ‘train‐test contamination’ has occurred that is, the same information that guides BINge clustering is also being assessed by the evaluation. When considering clustering outcomes that do not make use of the species' own reference genome, we observe a trend where the use of multiple related species' genomes results in greater locus cluster accuracy when compared to the use of any singular genome. This suggests that BINge is synthesising the information obtained from each individual genome to derive more accurate insight into gene orthology. In cases where multiple related genomes do not exist, we still observe that BINge maintains highly accurate clustering even when only a single related species' reference genome is available. This may provide cost‐saving benefits for researchers studying non‐model organisms which lack an assembled genome. Moreover, the agnosticism with which BINge handles the source of gene transcripts also facilitates flexible and cost‐saving experimental designs for example, the combination of species with reference genome availability alongside species which only have *de novo* transcriptome availability.

With respect to the amount of evolutionary distance that can be present between species whilst maintaining accurate clustering outcomes, our evaluations suggest that BINge is well suited to cluster transcripts from organisms within the same Genus (e.g., across citrus species) or Family classification (e.g., between mouse and rat). Species in the same Order also cluster well (e.g., between human and mouse or rat). Evolutionary separation to the Phylum level (e.g., between zebrafish and humans) reduces the reliability of results and is probably unsuitable for the application of BINge clustering; MMseqs2 similarly struggles in this scenario. It is likely that a DGE analysis should not be conducted between species with such large evolutionary separation for this and a variety of other reasons.

## Author Contributions

Z.S. designed the study under supervision of all other authors. Z.S. implemented the software, wrote the documentation, performed the benchmarking and evaluation study, and wrote the manuscript. All authors reviewed and edited the manuscript.

## Conflicts of Interest

The authors declare no conflicts of interest.

## Supporting information


**Figure S1:** A depiction of how a chimera may appear in a component during BINge's clustering by co‐occurrence module.

## Data Availability

All genome and sequence datasets are available in public databases through their accessions as indicated in the ‘Materials and Methods’ section or in Table S1. The BINge software is freely available under GPL‐3.0 licence at https://github.com/zkstewart/BINge.
