# Supplementary material for: BINge: Multispecies Ortholog Clustering for Differential Gene Expression Analyses
Source: Mol Ecol Resour. 2026 Apr 27;26:e70137. doi: 10.1111/1755-0998.70137 (PMC13112135; doi:10.1111/1755-0998.70137)
Supplement: Supplementary file 1 — Figure S1: A depiction of how a chimera may appear in a component during BINge's clustering by co‐occurrence module. [file MEN-26-e70137-s001.zip › FigureS1.docx]

# Supplementary Figures


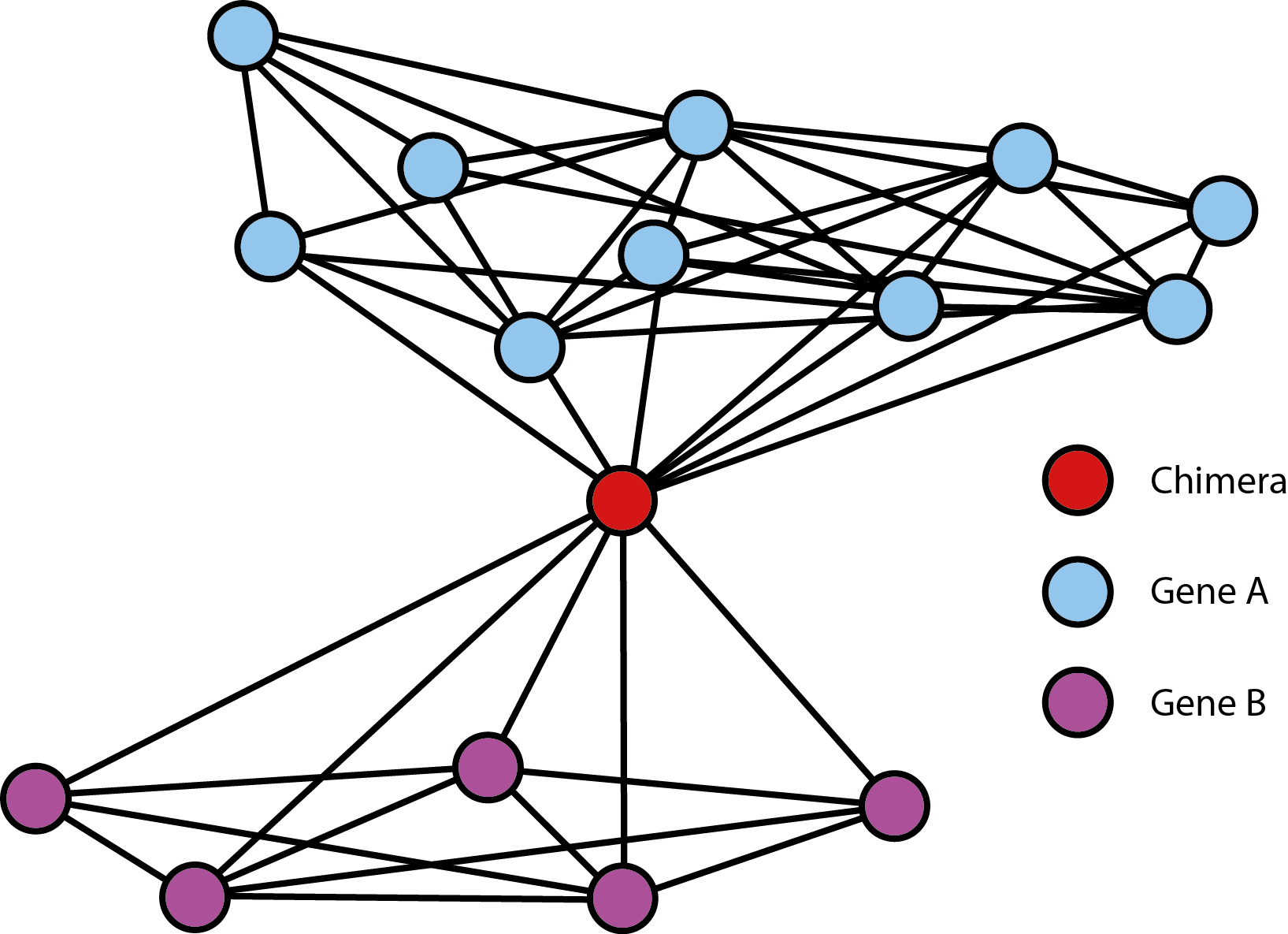


**Supplementary Figure 1.** A depiction of how a chimera may appear in a component during BINge’s clustering by co-occurrence module. The chimeric transcript, represented as a red node, erroneously contains genomic sequence from both genes A and B and subsequently aligns over exons that occur within those genes. Its deletion would leave no edges connecting the gene A and gene B subgraphs.
